# The Comparative Effectiveness of Education Modalities on Patient Adherence in Breast Cancer Survivors: A Systematic Review and Network Meta-Analysis

**DOI:** 10.3390/healthcare14091179

**Published:** 2026-04-28

**Authors:** Patricia Martínez-Miranda, María Jesús Muñoz-Fernández, Abel Rosales-Tristancho, Cristina García-Muñoz

**Affiliations:** 1 Departamento de Ciencias Clínicas y de la Salud, Universidad Loyola Andalucía, 41704 Seville, Spain; pmartinez@uloyola.es; 2 CTS 1110: Understanding Movement and Self in Health from Science (UMSS) Research Group, 41009 Andalusia, Spain; cgmunoz@us.es; 3 Department of Physiotherapy, Francisco Maldonado University School, 41640 Seville, Spain; 4 Department of Applied Economics I, Universidad de Sevilla, Avenida de Ramón y Cajal, 1, 41018 Seville, Spain; artristancho@us.es; 5 Department of Physiotherapy, University of Seville, 41009 Seville, Spain

**Keywords:** adherence, breast cancer, education, quality of life, adherence

## Abstract

Background: Educational interventions are central to breast cancer survivorship care, yet adherence may vary depending on delivery modality. Objective: To compare the effectiveness of face-to-face, online, telephonic, and mixed educational modalities on patient adherence among breast cancer survivors. Methods: A systematic review of randomized controlled trials and Bayesian network meta-analysis were conducted following PRISMA 2020 guidelines. Randomized controlled trials evaluating educational interventions in breast cancer survivors were included. Methodology quality of included studies was assessed using the RoB-2 tool. Pairwise meta-analyses using random-effects models estimated Odds Ratios (ORs) for adherence. A Bayesian network meta-analysis synthesized direct and indirect evidence, and treatment rankings were calculated using SUCRA values. Results: Eleven trials comprising 963 participants were included. In pairwise meta-analysis, no modality demonstrated statistically significant superiority over usual care: face-to-face (OR 0.79; 95% CI 0.44–1.41), mixed (OR 0.42; 95% CI 0.07–2.37), online (OR 0.90; 95% CI 0.49–1.68), and telephonic (OR 0.57; 95% CI 0.18–1.78). The network meta-analysis confirmed the absence of statistically significant differences across modalities. SUCRA rankings suggested that usual care (76.7%) and online modalities (73.1%) had the highest probability of being among the best-performing strategies, followed by face-to-face (51.9%), telephonic (25.4%), and mixed (23.0%). Conclusions: No educational modality demonstrated superior adherence compared to usual care. Delivery format alone may not determine engagement in breast cancer survivorship programs. Decisions should prioritize feasibility and patient preference.

## 1. Introduction

Breast cancer is the most frequently diagnosed cancer among women worldwide and represents a major public health challenge. According to the most recent global data, approximately 2.3 million new cases and 670,000 deaths were estimated in 2022, reflecting both its increasing epidemiological burden and the growing number of women living beyond diagnosis [1,2]. Notably, substantial disparities exist between high-income and low- and middle-income countries in terms of incidence, mortality, and access to cancer care, which may influence survivorship outcomes and the implementation of supportive interventions [1,2].

Advances in early detection and treatment have significantly expanded the population of breast cancer survivors, shifting the focus beyond tumor control toward survivorship outcomes. Many survivors experience persistent physical and psychosocial sequelae, including fatigue, pain, and psychological distress, which may negatively affect quality of life and daily functioning [3,4]. Current survivorship care guidelines emphasize the need for coordinated and comprehensive follow-up incorporating symptom management, rehabilitation, health promotion, and patient education as essential components of long-term recovery and well-being [3].

Although different studies have evaluated educational and rehabilitation interventions in this population, most have primarily focused on clinical outcomes such as quality of life, symptom reduction, and psychological well-being [5,6]. Consequently, adherence has often been treated as a secondary outcome, despite its central relevance for real-world implementation and sustainability of survivorship programs.

Adherence in this context refers to participants’ engagement with the educational intervention, including attendance, completion of sessions, and sustained participation over time, rather than medication-taking behavior. It represents a key determinant of the effectiveness, feasibility, and sustainability of complex health interventions, as insufficient engagement may limit their real-world impact [7]. However, its definition and measurement vary across studies, representing an important source of heterogeneity.

Among the factors that may influence adherence to educational programs for breast cancer survivors is the delivery channel of the intervention, which may include online, telephonic, face-to-face, or mixed approaches combining more than one modality [5,6]. Face-to-face interventions may facilitate personalization and therapeutic interaction, whereas digital and telehealth-based strategies may enhance accessibility, flexibility, and scalability of supportive care interventions. Mixed approaches may combine these advantages but may also increase intervention complexity [8,9]. In addition, differences in healthcare systems, access to digital resources, and sociocultural factors across geographical contexts may influence patient engagement and intervention uptake, potentially limiting the generalizability of findings across settings [7,9]. However, it remains unclear whether delivery modality itself exerts a meaningful impact on adherence, or whether engagement is primarily determined by other structural and contextual components of the intervention [6,7].

In this regard, network meta-analysis provides a methodological framework that enables the simultaneous comparison of multiple educational modalities by integrating direct and indirect evidence, offering a structured approach to evaluate their comparative effectiveness in terms of adherence and engagement. Beyond conventional pairwise meta-analysis, this approach allows for the estimation of relative effects between interventions that have not been directly compared, facilitates the ranking of interventions, and supports more comprehensive decision-making when multiple alternatives coexist [10,11]. Therefore, this study aimed to compare adherence across different educational delivery modalities in breast cancer survivors using a network meta-analysis approach.

## 2. Materials and Methods

### 2.1. Data Sources and Search Strategy

This systematic review and network meta-analysis was conducted and reported in accordance with the Preferred Reporting Items for Systematic reviews and Meta-Analyses (PRISMA) 2020 statement and the PRISMA 2020 for Abstracts guidelines [12]. The protocol was prospectively registered in the Open Science Framework (OSF) registry under the identifier 10.17605/OSF.IO/HMUQX.

A comprehensive literature search was independently performed by two reviewers (PMM and MJMF) across PubMed, Web of Science, Cumulative Index of Nursing and Allied Literature Complete (CINAHL), and Scopus, covering all records from database inception until 16 January 2026. Search strategies combined the terms Breast Neoplasms, Breast Cancer, Health Education, and Quality of Life, applying Boolean operators tailored to each database. Filters were applied according to the study design, focusing exclusively on randomized controlled trials (RCTs). More detailed search information is available in Supplementary File S1.

### 2.2. Research Question and Study Selection

The eligibility criteria were defined according to the PICOS framework (Population, Intervention, Comparator, Outcomes, and Study design). The inclusion criteria were:

− Population: Adult women diagnosed with breast cancer who had completed primary treatment (surgery, chemotherapy, or radiotherapy) (breast cancer survivors).
− Intervention: Any educational modality designed to improve quality of life.
− Comparators: A different educational modality or a passive control group, also aimed at improving quality of life.
− Outcomes: Patient’s adherence to the intervention.
− Study design: RCTs that explicitly reported adherence rates or provided sufficient data to estimate them indirectly.

The exclusion criteria were:

− Studies in which the comparator used the same educational modality as the intervention.
− Studies whose intervention combined education with another therapy.

### 2.3. Data Extraction

The reviewers who conducted the literature search (PMM and MJMF) also screened titles and abstracts to identify potentially eligible studies, following the PRISMA 2020 framework [12]. Duplicates were removed using Mendeley Desktop (version 1.19.8). Full-text articles were then assessed to determine compliance with the predefined eligibility criteria. Any disagreements were resolved by consulting a third reviewer (CGM).

For the qualitative synthesis, data were extracted and summarized regarding the following the PICOs model:

− Study characteristics: Author names and year of publication.
− Population: Total number of participants, sample size in experimental and control groups, mean age, cancer stage, and treatment history.
− Interventions: Educational modality used in both experimental and control groups, description of the program, frequency and duration of sessions, and overall intervention length.
− Outcomes: Adherence rates, dropout rates, reasons for withdrawal, and reported adverse events.

All extracted data were organized in structured tables to facilitate qualitative comparison across studies. For the quantitative synthesis, relevant variables were compiled in Microsoft Excel and prepared for statistical analysis in R.

### 2.4. Data Analysis

The primary outcome of this study was adherence to the educational intervention. Adherence was defined as participants’ engagement with the intervention, including measures such as session attendance, completion rates, and continued participation, as reported in the original studies.

Data synthesis commenced with an overview of the primary outcome: patient adherence among breast cancer survivors. For studies deemed sufficiently similar in design and population, a quantitative synthesis was conducted, beginning with pairwise meta-analysis. For this analysis of event data, we utilized the Odds Ratio (OR) as the summary measure of effect, reported with 95% confidence intervals. All statistical pooling was performed on the log-odds scale (i.e., the logarithm of the OR). For the meta-analysis estimation, a random-effects model was employed using the Mantel–Haenszel method for the pooled effect estimation and the DerSimonian–Laird estimator for the between-study variance [13]. Statistical heterogeneity was evaluated using the I2 statistic. Forest plots were generated to visualize these comparisons. This component of the analysis was performed in R (version 4.5.2) through the open-source integrated development environment RStudio (version 2025.09.2). Specifically, all statistical analyses were conducted using the meta package in R [14].

Following the pairwise meta-analysis, a network meta-analysis (NMA) was performed to synthesize the comprehensive evidence on the comparative effectiveness of the different educational modalities [15]. This approach integrated evidence from direct trials with indirect evidence derived via common comparators. Thus, the NMA framework generated relative effect estimates for all possible intervention comparisons, including those pairs not directly evaluated in any single study [15].

A hierarchical Bayesian model, implemented through Markov Chain Monte Carlo Simulations (MCMC), was employed to conduct the NMA [16,17]. A random-effects model was chosen to appropriately account for expected heterogeneity (i.e., within-study and between-study variability). The model convergence was assessed by calculating the Potential Scale Reduction Factor (PSRF). An acceptable convergence was defined as a PSRF value approaching 1 (and specifically, lower than 1.05). The outputs of the NMA included relative effect estimates (reported as ORs) for all pairwise comparisons, along with probabilistic treatment rankings presented as the SUCRA (Surface Under the Cumulative Ranking Curve) scores. The SUCRA score represents the cumulative probability that a treatment is among the best, with values closer to 1 (or 100%) indicating a higher likelihood of it being the most effective treatment, and values approaching 0 indicating a higher likelihood of it being least effective. The NMA was implemented in R and RStudio, using the gemtc and dmetar packages, by [ART] and subsequently reviewed by the full research team [16,17,18,19].

Furthermore, if substantial heterogeneity was identified, we explored its potential sources through subgroup analysis or meta-regression. These exploratory analyses were based on pre-specified study-level covariates. The feasibility of this analysis was contingent upon the availability and consistent reporting of these variables within the included studies.

## 3. Results

### 3.1. Study Selection

**Figure 1:**
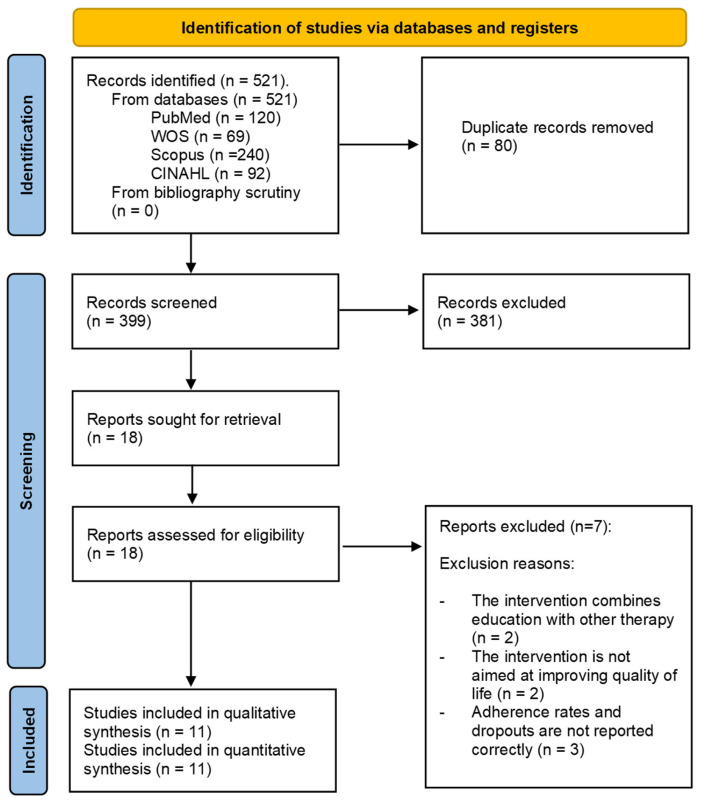
PRISMA flow diagram of study selection. A total of 11 randomized controlled trials were included in the final analysis.

The selection process was performed in accordance with the PRISMA recommendations. A total of 479 records were identified through database searching (PubMed, Web of Science, Scopus, and CINAHL). After removing duplicates (n = 80), 399 records were screened by title and abstract. Of these, 381 records were excluded, and 18 full-text reports were assessed for eligibility. Seven reports were excluded with reasons, and 11 studies [20,21,22,23,24,25,26,27,28,29,30] were included in the qualitative and quantitative synthesis. A list of studies excluded after full-text review is provided in Supplementary File S2. Figure 1 and Supplementary File S2 provide detailed information on the study selection process and the full-text exclusions.

### 3.2. Risk of Bias Assessment: Risk of Bias Tool 2 (ROB-2)

**Figure 2:**
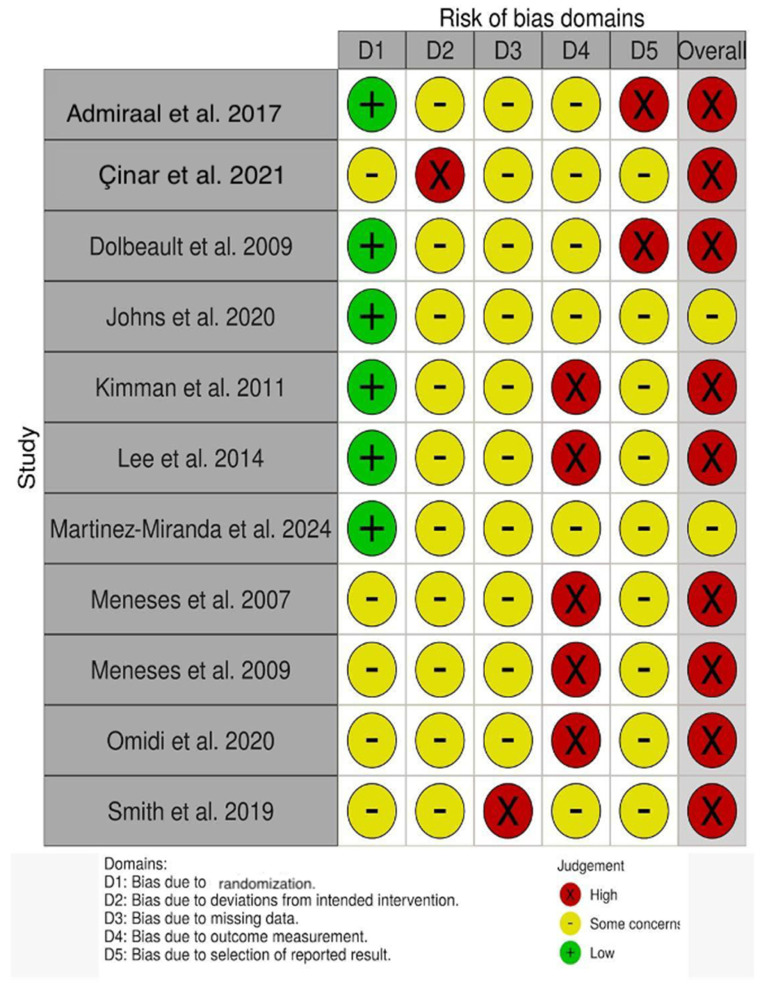
Risk of bias assessment (RoB-2). Most studies were rated as high risk of bias, mainly due to concerns in outcome measurement and lack of blinding. Meneses et al. [20]; Smith et al. [21]; Admiraal et al. [22]; Kimman et al. [23]; Dolbeault et al. [24]; Meneses et al. [25]; Martínez-Miranda et al. [26]; Çinar et al. [27]; Lee et al. [28]; Omidi et al. [29]; Johns et al. [30].

Overall, the ROB-2 assessment indicated that most included trials were at high risk of bias, mainly due to concerns related to outcome measurement and, in some cases, selective reporting. Blinding was generally not feasible, and outcomes were predominantly self-reported, which limits confidence in the estimated effects. A small number of studies were judged to have only some concerns overall, reflecting comparatively stronger methodological safeguards. Therefore, the available evidence should be interpreted with caution considering these methodological limitations. Figure 2 shows the extended results.

### 3.3. Description of the Selected Studies

Table 1 summarizes the characteristics of the 11 randomized controlled trials [20,21,22,23,24,25,26,27,28,29,30] included in the qualitative and quantitative synthesis, comprising a total of 963 breast cancer survivors who had completed primary treatment. Sample sizes ranged from 49 participants [26] to 261 participants [20]. Mean age generally ranged from early 40s [28] (41.5 ± 6.3 years) to late 50s [30] (58.70 ± 10.65 years).

Most trials included women diagnosed with stage I–III breast cancer, although cancer stage was inconsistently reported in some studies. Treatment history typically included surgery alone or combined with radiotherapy and/or chemotherapy, and in some cases hormonal therapy was reported. However, time since diagnosis and detailed treatment trajectories were not uniformly described across trials.

All included studies evaluated structured educational interventions aimed at improving survivorship outcomes, including quality of life, symptom self-management, coping strategies, and health behavior modification. Four educational delivery modalities were identified: face-to-face interventions (four studies [23,24,29,30]), online interventions (six studies [21,22,26,27,28,29]), telephonic interventions (one study [23]), and mixed interventions combining more than one modality (two studies [20,25]).

Face-to-face interventions were delivered in person, generally in group formats led by psychologists, oncology social workers, physiotherapists, or trained health professionals. Intervention duration ranged from brief formats such as two 2.5 h sessions [24] to structured multi-session programs delivered weekly over several weeks [24,30]. Intervention content typically included cognitive–behavioral strategies, stress management, communication skills, coping strategies, fear of cancer recurrence management, lymphedema self-management, and education on survivorship-related symptoms.

Online interventions were delivered through web-based platforms, videoconferencing systems, mobile applications, or social network-based tools. Formats included fully asynchronous web-based psychoeducational programs [22], videoconference-based group education [26], smartphone application-based programs with continuous access and reminders [27], and web-based self-management programs tailored according to behavioral stages [28]. One intervention used a social network-based educational program delivered through a Telegram™ channel [29]. Intervention duration ranged from three weeks to eighteen weeks, and some programs incorporated optional professional contact or digital support.

Telephonic modality was evaluated in one trial [23] and consisted of structured nurse-led telephone consultations conducted over an 18-month follow-up period. These calls focused on monitoring physical and psychological symptoms, discussing treatment-related side effects, supporting hormonal therapy adherence, and providing opportunities for open discussion and guidance.

Mixed interventions combined more than one delivery modality, typically integrating face-to-face educational sessions with structured telephone follow-up. Two trials [20,25] implemented psychoeducational programs that blended in-person sessions with telephonic reinforcement and supplementary written materials. These interventions aimed to strengthen continuity of support by combining different communication channels throughout the survivorship period.

Comparators across the included studies were usual care. In most trials, usual care corresponded to standard oncological follow-up without additional structured educational intervention. In some studies, this included minimal-support approaches such as waiting-list controls, routine clinical follow-up, or the provision of written educational materials. In several trials, participants allocated to the control group were offered access to the educational content after completion of the study period.

Adherence rates varied across studies, ranging from 55.74% in the online intervention by Smith et al. [21] to 100% in two trials [25,27]. Dropout rates ranged from 0% to 44.26%, with the highest attrition observed in the online intervention evaluated by Smith et al. [21]. Across the included studies, adherence rates were generally high, with most interventions reporting values above 80%, regardless of the delivery modality. However, greater variability was observed in online interventions, where both the lowest adherence and the highest dropout rates were reported. Face-to-face and mixed modalities tended to show more consistent adherence patterns, although differences between intervention and control groups were often small. Overall, these findings suggest that factors beyond delivery format may play a more relevant role in determining adherence in breast cancer survivorship programs.

Reported reasons for dropout were heterogeneous and included competing work or family responsibilities, logistical barriers such as living in other cities or difficulties attending sessions, dissatisfaction with group allocation, loss of contact, health deterioration, cancer recurrence or metastasis, development of other cancers, and incomplete questionnaire data. Adverse events were rarely reported across studies, and none of the trials identified major safety concerns attributable to participation in the educational interventions.

### 3.4. Pairwise Meta-Analysis

**Figure 3:**
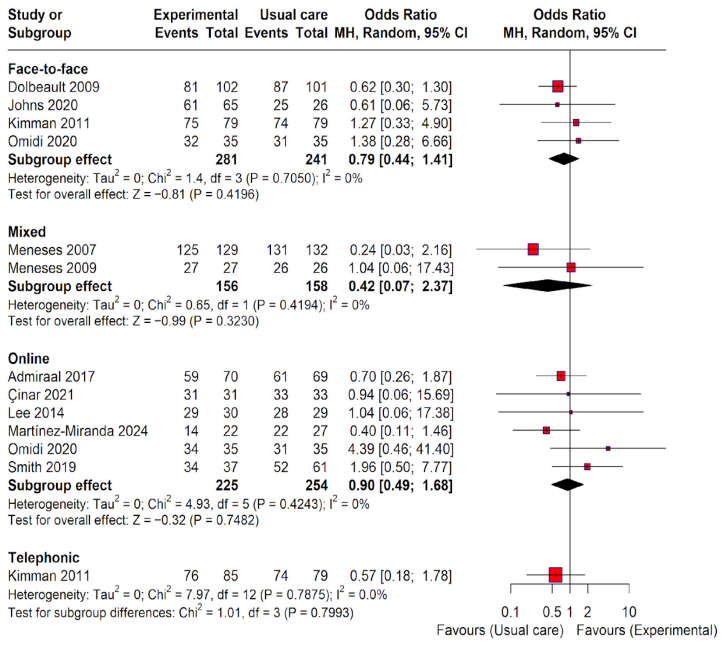
Forest plot of pairwise meta-analyses for all intervention modalities versus usual care. Squares (shown in red) represent the effect size (odds ratio) for each individual study, with the size of the square proportional to the study weight. Horizontal lines indicate 95% confidence intervals. Black diamonds represent pooled effect estimates for each subgroup and overall, with their width corresponding to the 95% confidence interval. The vertical line at odds ratio (OR) = 1 indicates no effect. Values less than 1 favour the experimental group, whereas values greater than 1 favour usual care. Analyses were performed using a random-effects model. Heterogeneity was assessed using the I^2^ statistic. Meneses et al. [20]; Smith et al. [21]; Admiraal et al. [22]; Kimman et al. [23]; Dolbeault et al. [24]; Meneses et al. [25]; Martínez-Miranda et al. [26]; Çinar et al. [27]; Lee et al. [28]; Omidi et al. [29]; Johns et al. [30].

A pairwise meta-analysis was conducted to evaluate the effect of the different educational modalities’ adherence compared to usual care (Figure 3). The results are presented by intervention modality subgroups. Following the Cochrane Handbook recommendations, in studies with multiple experimental arms of the same modality, the groups were combined to obtain a single pairwise comparison against the usual care group and avoid the double-counting of the unit of analysis. The analysis included a total of 13 direct comparisons (representing a total sample of 1365 observations, with 747 in the intervention groups and 618 in the control group). In studies with zero cell frequencies (100% adherence or 0% adherence), a continuity correction of 1 unit was applied to ensure the stability of the estimates. In our case, only studies with 100% adherence appeared.

In the subgroup analysis, no specific modality reached statistical significance compared to usual care in terms of direct evidence: face-to-face (OR 0.79; 95% CI 0.44 to 1.41), mixed (OR 0.42; 95% CI 0.07 to 2.37), online (OR 0.90; 95% CI 0.49 to 1.68), and telephonic (OR 0.57; 95% CI 0.18 to 1.78). An Odds Ratio (OR) below 1 indicates that the advantage (odds) of exhibiting adherence versus non-adherence is lower in the experimental group than in the usual care group. In practical terms, this reflects a lower propensity for adherence in the intervention groups across all analyzed modalities. However, since all confidence intervals included the value of 1, the results are considered non-significant. The test for subgroup differences confirmed that the type of treatment employed did not significantly influence the observed effect (*p*-value = 0.8). Furthermore, no statistical heterogeneity was detected among the studies within each analyzed subgroup (I^2^ = 0%).

To assess the robustness of the findings, sensitivity analyses using the leave-one-out method were conducted within the intervention subgroups with at least three comparisons (face-to-face and online). For both modalities, the iterative omission of individual studies did not significantly alter the subgroup-specific estimates, and the OR values remained non-significant across all iterations. Specifically, in the face-to-face subgroup, the heterogeneity remained at I^2^ = 0%, showing high stability in the effect size despite the omission of any single study (Table 2). Similarly, the online subgroup showed consistent results: although the omission of certain comparisons introduced minor variations in the heterogeneity index (up to I^2^ = 18.9%), the overall direction and lack of statistical significance remained unchanged (Table 3). For the mixed modality subgroup, sensitivity analysis was not performed as the omission of one study would eliminate the comparative structure necessary for meta-analytical synthesis. These results confirm that direct evidence is not driven by any single influential study, thereby supporting the overall stability and reliability of the findings for each intervention modality.

### 3.5. Network Meta-Analysis

A network meta-analysis (NMA) was performed within a Bayesian framework to simultaneously compare all intervention modalities. The analysis employed a random-effects model, with parameter estimation conducted via Markov Chain Monte Carlo (MCMC) methods. Model convergence was confirmed by ensuring all potential scale reduction factors (PSRFs) were close to 1.0. As was done in the pairwise meta-analyses, a continuity correction of 1 unit was applied to treatments with 100% adherence to maintain model stability.

**Figure 4:**
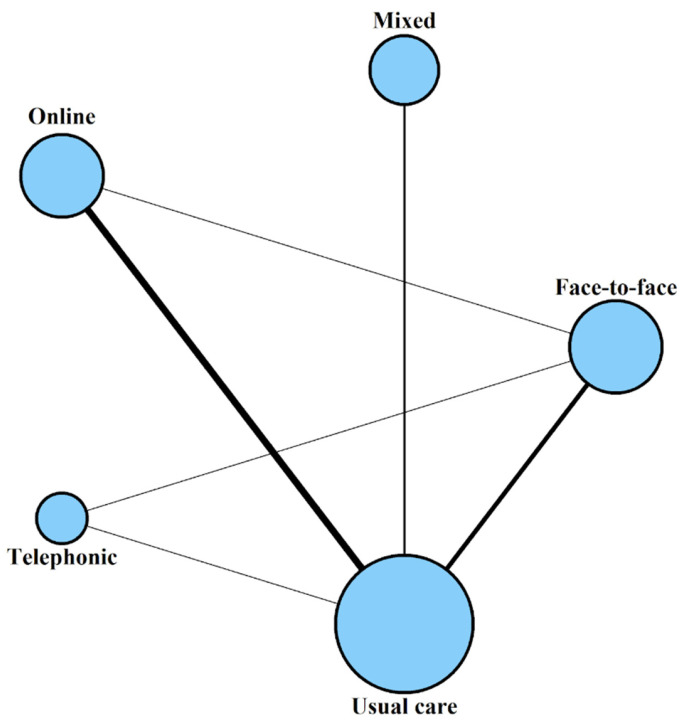
Network plot of direct comparisons between intervention modalities.

The network structure is shown in Figure 4. Node size is proportional to the sample size of each treatment, while the thickness of each edge is proportional to the number of comparisons between each pair of treatments. The network comprises five intervention modalities connected via direct comparisons. The group with the largest volume of evidence was usual care, with a total of 618 participants, followed by face-to-face with 281 participants, online with 225, mixed with 156, and telephonic with 85 participants. The most frequent comparisons correspond to the relationship between usual care and online, with a total of six studies, followed by usual care and face-to-face with four studies. The network connectivity is also supported by two three-arm trials (usual care vs. face-to-face vs. online, and usual care vs. face-to-face vs. telephonic).

Table 4 presents the descriptive statistics of the model through the quantiles of the posterior distribution for each intervention. This table allows for the observation of the median effect (50% quantile) and the dispersion of the results. The values corresponding to the 2.5% and 97.5% quantiles define the 95% credible interval (CrI) for each comparison group against usual care.

Regarding the odds of adherence, the face-to-face modality presents a median of 0.74, mixed 0.34, and telephonic 0.43. Online intervention shows a median of 0.98. All treatments showed a median OR less than 1, suggesting a general tendency toward a lower propensity for adherence compared to the usual care group. These values represent a point estimate of the relative effect magnitude for each modality within the network.

Table 5 shows the relative comparisons between all pairs of interventions in the network using a matrix of Odds Ratios (ORs) and 95% credible intervals (CrIs). An OR value greater than 1 indicates a higher advantage (odds) of adherence for the intervention in the column compared to the one in the row. Conversely, a value below 1 indicates that the intervention in the row has a higher advantage compared to the column.

The results of the indirect comparisons between the different treatment modalities showed no statistically significant differences, as all credible intervals included the null value (OR = 1). Online intervention showed a clinical tendency toward higher adherence over both the mixed (2.91; 95% CrI 0.41 to 28.99) and telephonic (2.31; 95% CrI 0.49 to 11.22) modalities. Additionally, a similar tendency was observed in favor of usual care over the telephonic modality (2.34; 95% CrI 0.58 to 9.23). In some cases, the width of the intervals precludes confirming the statistical superiority of one treatment over another.

**Figure 5:**
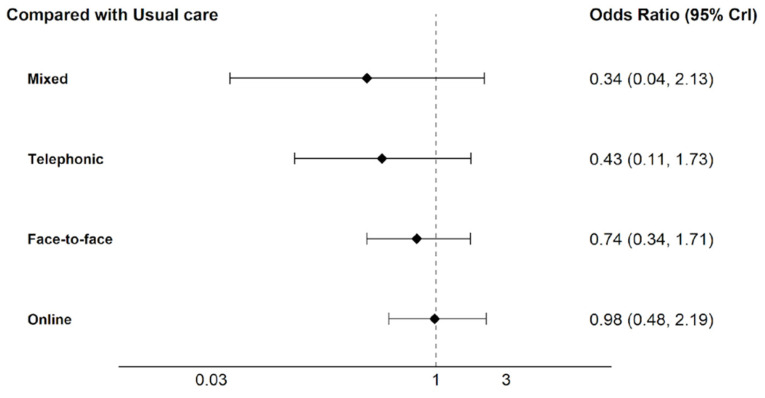
Forest plot of Odds Ratios for interventions versus usual Care.

Figure 5 presents the Odds Ratio estimates for each intervention modality compared with usual care. Due to the width of the credible intervals in certain comparisons, the horizontal axis is presented on a logarithmic scale, highlighting the network’s values to facilitate the visual interpretation of the estimates. Values below 1 indicate a trend in adherence favoring the usual care group.

The ranking analysis based on SUCRA (Table 6) revealed that usual care had the highest probability of being the best intervention (76.7%), followed by online modality (73.1%). The face-to-face modality (51.9%) occupied an intermediate position, while telephonic (25.4%) and mixed (23.0%) groups recorded the lowest scores.

**Figure 6:**
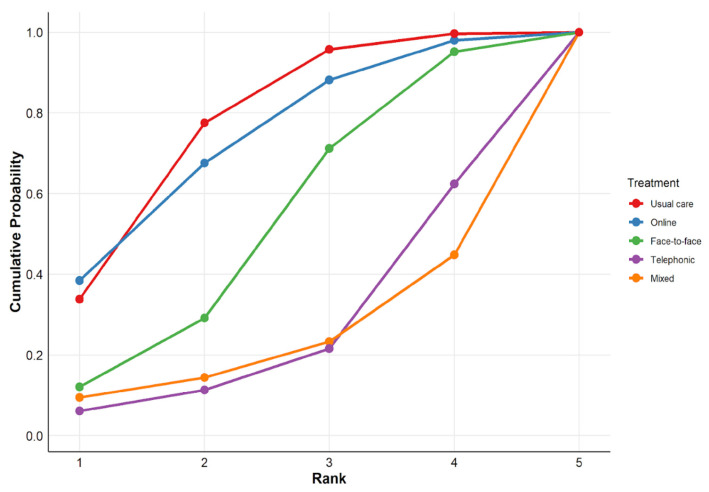
Cumulative ranking probability curves (SUCRA).

Figure 6 shows the cumulative probability curves for the evaluated treatments. The analysis of these curves allows for a visualization of which interventions are most likely to achieve the top rankings in terms of effectiveness. A steeper initial slope and a position toward the upper-left corner indicate a higher probability of effectiveness. Usual care and online modalities present the most dominant paths, rapidly accumulating the highest probability of occupying the top-ranking positions. In contrast, mixed and telephonic modalities show curves shifted significantly to the right, confirming their positions as the options with the lowest probability of being superior within the treatment network.

**Figure 7:**
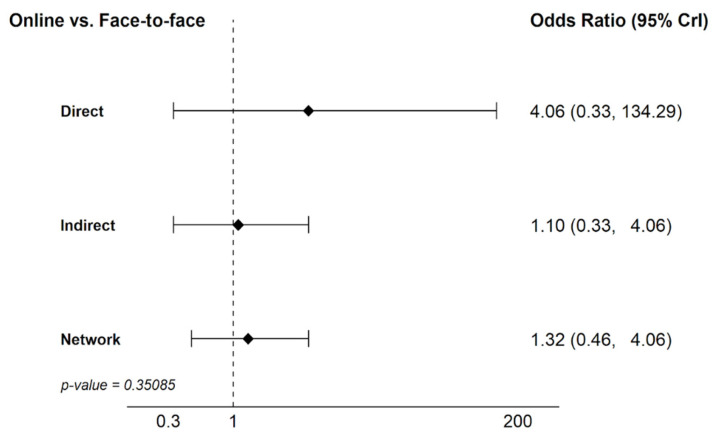
Node-splitting analysis for the assessment of inconsistency: online vs. face-to-face comparison.

To assess the reliability of the network meta-analysis results, an inconsistency analysis was performed using the node-splitting method. This approach was focused on the comparison between face-to-face and online modalities, as it was the loop in the network featuring sufficient evidence to allow for a robust separation of direct and indirect estimates. The results in Figure 7 showed no significant differences between the direct and indirect estimates (*p*-value = 0.35). The direct estimate was 4.06 (95% CrI 0.33 to 134.29) and the indirect estimate was 1.10 (95% CrI 0.33 to 4.06), both being statistically consistent with the overall network estimate.

**Figure 8:**
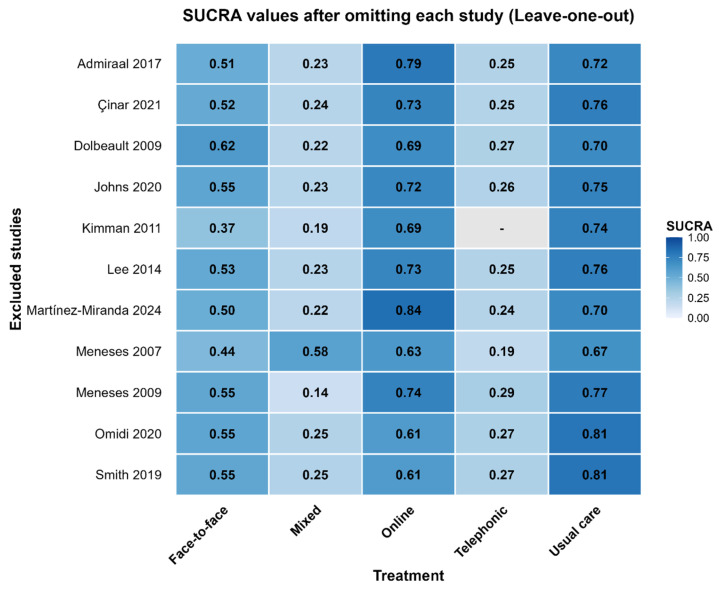
Leave-one-out sensitivity analysis of treatment rankings. Meneses et al. [20]; Smith et al. [21]; Admiraal et al. [22]; Kimman et al. [23]; Dolbeault et al. [24]; Meneses et al. [25]; Martínez-Miranda et al. [26]; Çinar et al. [27]; Lee et al. [28]; Omidi et al. [29]; Johns et al. [30].

The robustness of the NMA was evaluated through a leave-one-out sensitivity analysis (Figure 8). All new models reached optimal convergence, ensuring the reliability of the recalculated estimates. The most prominent finding was the high stability of the primary results: usual care and online modalities consistently emerged as the most effective interventions across almost all iterations. While the mixed modality showed some sensitivity to the removal of Meneses (2007) [20], the overall hierarchy of the network remained largely unchanged. These findings confirm that the conclusions of this study are robust and are not driven by any individual outlier study. It should be noted that the absence of the telephonic modality when excluding Kimman (2011) [23] is was due to this treatment being exclusively evaluated in that single trial.

## 4. Discussion

This network meta-analysis found no statistically significant differences in adherence among face-to-face, online, telephonic, and mixed educational modalities and usual care in breast cancer survivors. Across both pairwise and network analyses, effect estimates were consistently non-significant. Although SUCRA rankings suggested that usual care and online interventions had a higher probability of being among the best-performing options, these findings must be interpreted cautiously due to the absence of statistical superiority.

Several factors may explain the absence of statistically significant differences between educational modalities. First, the included interventions were highly heterogeneous in terms of content, intensity, duration, and level of professional support, which may have diluted potential modality-specific effects. Second, adherence was inconsistently defined and measured across studies, introducing additional variability and limiting comparability. Third, patient engagement in survivorship programs is likely influenced by contextual and individual factors, such as motivation, health status, digital literacy, and competing life demands, rather than by the delivery modality alone. Taken together, these findings suggest that the effectiveness of educational interventions in promoting adherence may depend more on their design and contextual adaptation than on the format through which they are delivered.

These findings are consistent with the conceptual framework proposed by the World Health Organization as well as with previous empirical evidence on adherence behavior [7,31,32,33]. The World Health Organization conceptualizes adherence as a multidimensional construct shaped by patient-related, therapy-related, condition-related, health system, and socioeconomic domains. According to this model, adherence is determined by the interaction of multiple factors rather than by isolated structural characteristics of an intervention [7].

Empirical evidence further supports this perspective. Prior research indicates that adherence to health-related interventions is influenced by patient characteristics, intervention demands, psychological and contextual determinants, and system-level factors [31,32,33]. Therefore, within this multifactorial framework, adherence cannot be attributed to a single structural characteristic of an intervention, such as its delivery channel.

In line with this perspective, several additional factors may be particularly relevant in shaping adherence in breast cancer survivorship programs. Patient-related aspects such as motivation, perceived relevance of the intervention, psychological status, and digital literacy may strongly influence engagement. Intervention-related characteristics, including personalization, interactivity, intensity of follow-up, and the presence of professional support, may also play a key role. Furthermore, contextual factors such as social support, accessibility, competing life demands, and healthcare system organization may further impact adherence. These considerations reinforce the need to move beyond a modality-centered approach and toward more tailored, patient-centered survivorship interventions.

### 4.1. Research and Clinical Implications

From a clinical perspective, the absence of superiority across modalities suggests that selecting an educational delivery format based solely on expectations of higher adherence may not be justified. Since no modality demonstrated a clear advantage, decisions regarding implementation may reasonably prioritize feasibility, accessibility, scalability, cost, and patient preference.

The relatively favorable SUCRA positioning of online interventions is coherent with global strategies promoting digital health accessibility and scalability. However, engagement in digital interventions appears to depend more on structured support and personalization than on digital delivery per se. Thus, educational content design, behavioral reinforcement mechanisms, and integration into survivorship pathways may represent more relevant targets for optimization than modality classification [3,7].

Future research should focus on standardizing the definition and measurement of adherence in educational interventions, as well as identifying the key behavioral and contextual factors that influence patient engagement. Additionally, further high-quality randomized controlled trials with consistent reporting are needed to better understand the role of intervention components beyond delivery modality. The integration of personalized and digitally supported approaches may also represent a promising direction to enhance adherence in breast cancer survivorship care.

Finally, research should explore the long-term sustainability of adherence beyond intervention completion, particularly given the chronic and long-term nature of breast cancer survivorship.

### 4.2. Limitations

Several limitations should be acknowledged when interpreting the findings of this network meta-analysis.

First, the overall methodological quality of the included trials was limited. According to the ROB-2 assessment, most studies were judged to have a high risk of bias, particularly in the measurement of adherence outcomes, which were predominantly self-reported.

Second, adherence was operationalized heterogeneously across studies (e.g., attendance rates, percentage of completed sessions, and dropout reasons), which limits comparability and may affect the precision of pooled estimates.

Third, clinical heterogeneity was present. Information regarding cancer stage, time since treatment completion, symptom burden, and concurrent therapies was inconsistently reported, restricting the possibility of conducting subgroup analyses to explore potential effect modifiers.

Fourth, certain modalities were underrepresented within the network. In particular, the telephonic intervention was evaluated in a single trial, limiting the robustness and stability of comparative estimates for this modality. Although sensitivity analyses suggested overall network stability, some comparisons were supported by sparse data and wide credible intervals.

Finally, the relatively small number of included studies and the limited sample sizes within some modalities contributed to imprecision, as reflected by wide confidence and credible intervals. Therefore, the absence of statistically significant differences should not be interpreted as definitive evidence of equivalence across educational delivery formats.

## 5. Conclusions

This systematic review and network meta-analysis found no statistically significant differences in adherence among face-to-face, online, telephonic, and mixed educational modalities and usual care in breast cancer survivors. Based on the available evidence, these findings suggest that delivery modality alone is unlikely to be a decisive determinant of adherence in survivorship programs.

Clinical decisions regarding educational implementation should therefore consider contextual feasibility, patient preference, and resource availability rather than assuming inherent superiority of a particular format. Importantly, the results must be interpreted with caution given the methodological limitations of the included trials.

## Figures and Tables

**Table 1:** Data extraction.

**Face-to-Face Patient Education Modality**								
**Study**	**Population**		**Intervention**	**Comparation**	**Adherence Rates**	**Dropout Rates**	**Reason of Dropouts**	**Reported Adverse Events**
Dolbeault et al., 2009 [24]	N: 203 CG: 101 EG: 102	Mean age (years): 54.5 ± 9.3 Cancer stage: not reported Treatment history: not reported	Face-to-face educational program based on CBT principles: use of thought records, problem-solving, cognitive restructuring; communication training through role play, relaxation practice, and thematic discussions (e.g., cancer impact, body image, uncertainty, communication with loved ones). Led by two therapists (psychologists/psychiatrists) (2 h/session; 1 session/week; 8 weeks).	Usual care: Waiting-list control (no intervention during the study period).	N: 82.76% (168/203) EG: 79.41% (81/102) CG: 86.14% (87/101)	N: 17.24% (35/203) EG: 20.59% (21/102) CG: 13.86% (14/101)	Disappointment with the group (n = 7) Disappointment with the randomized allocation (n = 6) Work problems (n = 3) Family problems (n = 3) Other non-cancer health problems (n = 4) Cancer recurrence (n = 1) Other reasons (n = 5) Not reported (n = 6)	Not reported
Kimman et al., 2011 [23]	N: 158 CG: 79 EG: 79	Mean age (years): EG1: 55.3 ± 9; CG: 56.2 ± 10.7 Cancer stage: I: n = 139/158 (87.97%) IIa: 56/158 (35.44%) IIb: 16/158 (10.13%) III: 19/158 (12.03%) Unknown: 2/158 (1.27%) Treatment history: S: 22/158 (13.92%) S + RT: 148/158 (93.67%) S + CT: 12/158 (7.59%) S + RT + CT: 62/158 (39.24%)	GE1: Face-to-face educational program lead by psychologist. Content: treatment side effects, recurrence signs/symptoms, prostheses, fatigue (2.5 h/session; 2 sessions; delivered within 3 months after end of treatment).	Usual care: Standard oncological care in hospital; no additional education (follow-up: 18 months).	N: 94.3% (149/158) EG: 94.94% (75/79) CG: 93.67% (74/79)	N: 5.7% (9/158) EG: 5.06% (4/79) CG: 6.33% (5/79)	Patient request (n = 1) Missing questionnaires (n = 1) Other cancers (n = 3) Metastases (n = 3) Recurrence (n = 1)	Not reported
Omidi et al., 2020 [29]	N: 70 CG: 35 EG: 35	Mean age (years): EG1: 52.47 ± 10.62; CG: 50.23 ± 8.9 Cancer stage: I: 4/70 (5.71%) II: 41/70 (58.57%) III: 25/70 (35.71%) Treatment history: not reported	EG1: Face-to-face group education program (groups of five). Content: lymphedema self-management and one session on stress management strategies. (60–90 min/session; 5 sessions, twice weekly for 3 weeks).	Usual care: Standard lymphedema treatment. They also received a CD containing the educational materials after the study period.	N: 90% (63/70) EG: 91.43% (32/35) CG: 88.57% (31/35)	N: 10% (7/70) EG: 8.57% (3/35) CG: 11.43% (4/35)	Living in other cities and failure to complete treatment (n = 4) Absence in third and fourth sessions (n = 3)	Not reported
Johns et al., 2020 [30]	N: 91 CG = 26 EG1 = 33 EG2 = 32	Mean age (years): 58.70 ± 10.65; EG1: 59.84 ± 11.10; EG2: 57.53 ± 10.52; CG: 58.68 ± 10.49 Cancer stage: I: 38 (41.76%) II: 39 (42.86%) III: 14 (15.38%); Treatment history: S: 12 (13.19%) S + RT: 18 (19.78%) S + CT: 19 (20.88%) S + CT + RT: 42 (46.15%) 52.47 ± 10.62 months since diagnosis	EG1: Face-to-face group program based on Acceptance and Commitment Therapy (ACT), led by a doctoral-level provider trained in mindfulness/acceptance-based therapies (10–12 participants/group). Content: coping with fear of cancer recurrence using acceptance, cognitive defusion, mindfulness, and perspective-taking exercises (6 sessions; 2 h/session; 1 session/week; 6 weeks). EG2: Face-to-face group survivorship education program led by masters-level oncology social workers (10–12 participants/group). Content: symptom management and health habits (6 sessions; 2 h/session; 1 session/week; 6 weeks).	Usual care and an educational booklet (6 weeks).	(EG1 vs. CG) N: 93.22% (55/59) EG: 90.91% (30/33) CG: 96.15% (25/26) (EG2 vs. CG) N: 96.55% (56/58) EG: 96.88% (31/32) CG: 96.15% (25/26)	(EG1 vs. CG) N: 6.78% (4/59) EG: 9.09% (3/33) CG: 3.85% (1/26) (EG2 vs. CG) N: 3.45% (2/58) EG: 3.13% (1/32)	Not reported	Not reported
**Online Patient Education Modality**								
**Study**	**Population**		**Intervention**	**Comparation**	**Adherence Rates**	**Dropout Rates**	**Reason of Dropouts**	**Reported Adverse Events**
Smith et al., 2019 [21]	N: 89 CG: 52 EG: 37	Mean age (years): 56.7 ± 8.7 EG: 56.1 ± 8.9 CG: 57.1 ± 8.6 Cancer stage: not reported Treatment history: not reported. Mean years since diagnosis 8.2 ± 6.6	Online educational program (Reimagine) including one synchronous group session (Adobe Connect) led by a trained facilitator and asynchronous web-based modules: videos, cognitive reframing, mind–body and relaxation exercises, and solution-focused stress management. (≈1 h introductory session; self-paced online activities; 18 weeks).	Usual care (not reported).	N: 70.49% (86/122) EG: 55.74% (34/61) CG: 85.25% (52/61)	N: 29.51% (36/122) EG: 44.26% (27/61) CG: 14.75% (9/61)	Lost contact, feeling too sick, lack of time	Not reported
Admiraal et al., 2017 [22]	N: 139 CG: 69 EG: 70	Mean age (years): EG: 53.1 ± 9.8; CG: 53.2 ± 8.5 Cancer stage: I: n = 63/139 (45.32%) II: 4/139 (2.88%) III: 71/139 (51.08%) Treatment history: not reported. Years since diagnosis—EG: 8.7 ± 2.1; CG: 8.7 ± 1.9	Online educational program (fully web-based, asynchronous) + optional contact with research psychologist (telephone/e-mail) (≥1 session first week, then flexible self-paced use; 12 weeks).	Usual care: Standard oncological care; no additional education (12 weeks).	N: 86.33% (120/139) EG: 84.29% (59/70) CG: 88.41% (61/69)	N: 13.67% (19/139) EG: 44.26% (27/70) CG: 14.75% (9/59)	Not reported	Not reported
Martínez-Miranda et al., 2024 [26]	N: 49 CG: 27 EG: 22	Mean age (years): EG: 49.21 ± 5.91; CG: 50 ± 8.04 Cancer stage: stage 0–III (reported; distribution not reported) Treatment history: not reported	Online educational program lead by a physiotherapist using a videoconference platform and online material to work at home (pain diary). In groups (10–15 participants/group). Content: pain neuroscience education, pain concept and mechanisms of pain, acute and chronic pain, pain as an individual experience, self-management and habits to improve quality of life related to pain (1 h/session; 2 sessions/week; 8 sessions; 1 month).	Usual Care. They received the content when the study ended (1 month).	N: 91.84% (45/49) EG: 90.91% (20/22) CG: 92.59% (25/27)	N: 8.16% (4/49) EG: 9.09% (2/22) CG: 7.41% (2/27)	Attended less than 50% of sessions (n = 2) Health reasons (n = 1) Unknown reason (n = 1)	None
Çinar et al., 2021 [27]	N: 64 CG: 33 EG: 31	Mean age (years): 45.7 ± 9; EG: 45.9 ± 8.3; CG: 45.5 ± 9.8 Cancer stage: I: n = 21/64 (32.81%) II: 24/64 (37.50%) III: 19/64 (29.69%) Treatment history: S: 40/64 (62.5%) CT: 49/64 (76.6%)	Mobile app-based educational program lead by a specialist nurse using a smartphone application with continuous access to educational modules, symptom diary, relaxation techniques (audio/video), reminders, and direct nurse counseling through the app. Content: breast cancer information, adjuvant endocrine therapy (EHT) side-effect management, coping strategies, relaxation and guided imagery exercises, and direct Q&A with the nurse (daily reminders and counseling through the app; continuous access; 12 weeks).	Usual care. After the study ended, the mobile app training content was provided as a written booklet (12 weeks).	N: 100% (64/64) EG: 100% (31/31) CG: 100% (33/33)	N: 0% (0/64) EG: 0% (0/31) CG: 0% (0/33)	None	None
Lee et al., 2014 [28]	N: 59 CG: 29 EG: 30	Mean age (years): EG: 41.5 ± 6.3; CG: 43.2 ± 5.1 Cancer stage: 0: n = 2/59 (3.39%) I: 23/59 (38.98%) II: 28/59 (47.46%) III: 6/59 (10.17%) Treatment history: S: n = 59/59 (100%) RT: 52/59 (88.14%) CT: 49/59 (83.05%)	Web-based self-management educational intervention (WSEDI) with tailored content according to Transtheoretical Model (TTM). Content: exercise and diet behavior enhancement in cancer survivors; educational modules tailored to stage of change (pre-contemplation, contemplation, preparation, action, maintenance) (1 brief introductory training session <30 min; encouraged to use ≥2 times/week; continuous access; 12 weeks).	Usual care and educational booklet program about exercise and diet recommendation for cancer survivors. (1 delivery of booklet at baseline; self-use; 12 weeks).	N: 96.61% (57/59) EG: 96.67% (29/30) CG: 96.55% (28/29)	N: 3.39% (2/59) EG: 3.33% (1/30) CG: 3.45% (1/29)	Busy (n = 1) Recurrence (n = 1)	Not reported
Omidi et al., 2020 [29]	N: 70 CG: 35 EG: 35	Mean age (years): EG2: 50.44 ± 8.81; CG: 50.23 ± 8.9 Cancer stage: I: n = 4/70 (5.71%) II: 41/70 (58.57%) III: 25/70 (35.71%) Treatment history: not reported	EG2: online education program using a social network-based program delivered via a dedicated Telegram™ channel. Content: lymphedema self-management and one session on stress management strategies. (20 audio and photo messages; twice weekly for 3 weeks).	Usual care: Standard lymphedema treatment. They also received a CD containing the educational materials after the study period.	N: 92.86% (65/70) EG: 97.14% (34/35) CG: 88.57% (31/35)	N: 7.14% (5/70) EG: 2.86% (1/35) CG: 11.43% (4/35)	Living in other cities and failure to complete treatment (n = 4) Failure to receive messages during the intervention, (n = 1)	Not reported
**Telephonic Patient Education Modality**								
**Study**	**Population**		**Intervention**	**Comparation**	**Adherence Rates**	**Dropout Rates**	**Reason of Dropouts**	**Reported Adverse Events**
Kimman et al., 2011 [23]	N: 164 CG: 79 EG: 85	Mean age (years): EG2: 55.5 ± 9; CG: 56.2 ± 10.7 Cancer stage: I: EG = 90/150 (60.0%); CG = 91/149 (61.1%) IIa: EG = 34/150 (22.7%); CG = 35/149 (23.5%) IIb: EG = 13/150 (8.7%); CG = 8/149 (5.4%) III: EG = 11/150 (7.3%); CG = 13/149 (8.7%) Unknown: EG = 2/150 (1.3%); CG = 2/149 (1.3%) Treatment history: S: EG = 14/150 (9.3%); CG = 15/149 (10.1%) S + RT: EG = 89/150 (59.3%); CG = 89/149 (59.7%) S + CT: EG = 8/150 (5.3%); CG = 7/149 (4.7%) S + RT + CT: EG = 39/150 (26.0%); CG = 38/149 (25.5%)	GE2: Synchronous telephonic educational calls by a nurse. Content: screening for physical/psychological symptoms, treatment side effects, hormonal therapy compliance, open discussion (duration per session not specified; 4 sessions; 18 months).	Usual care: Standard oncological care in hospital; no additional education (18 months).	N: 91.46% (150/164) EG: 94.94% (76/85) CG: 93.67% (74/79)	N: 8.81% (14/158) EG: 10.13% (8/79) CG: 6.33% (5/79)	Patient request (n = 4) Missing questionnaires (n = 3) Other cancers (n = 1) Metastases (n = 4) Recurrence (n = 1) Herceptin (n = 1)	Not reported
**Mixed Patient Education Modality**								
**Study**	**Population**		**Intervention**	**Comparation**	**Adherence Rates**	**Dropout Rates**	**Reason of Dropouts**	**Reported Adverse Events**
Meneses et al., 2007 [20]	N: 261 CG: 132 EG: 129	Mean age (years): 54.5 ± 11.58 Cancer stage: not reported. Treatment history: S: 261/261 (100%) RT: >180/261 (>69%) CT: 141/261 (54%) HT: 198/261 (76%)	Face-to-face and telephone-based educational support program. Content: coping with symptoms and side effects in the survival period; health habits. Educational support was reinforced through written materials and audiotapes (5 sessions/month (3 telephonic and 2 face-to-face); 60–90 min/session; 3 sessions face-to-face, 6 months).	Usual care: Attention-control telephone calls, rather than structured psychoeducational sessions. The participants received the educational content after the study ends (4 calls/month; 6 months).	N: 98.08% (256/261) EG: 96.90% (125/129) CG: 99.24% (131/132)	N: 1.92% (5/261) EG: 0.03% (4/129) CG: 0.76% (1/132)	Not reported	Not reported
Meneses et al., 2009 [25]	N: 53 CG: 26 EG: 27	Mean age (years): 53.58 ± 11.55 Cancer stage: I: 27/53 (50.9%) II: 26/53 (49.1%) Cancer treatment: S: 26/53 (49%) RT: 32/53 (60%) CT: 33/53 (62%); 8.6 ± 2.7 months since diagnosis	Mixed educational program: face-to-face sessions + telephonic sessions. Content: support focused on quality of life (physical, psychological, social, and spiritual well-being), symptom education (pain, fatigue, lymphedema), coping strategies, lifestyle behaviors, social issues (educational support: 60–90 min/face-to-face sessions; 3 face-to-face sessions; follow-up educational and support: ~30 min session; 2 face-to-face + 3 telephonic sessions; 6 months).	Usual care and monthly check-in telephone calls or visits from the research team (6 months).	N: 100% (53/53) EG: 100% (27/27) CG: 100% (26/26)	N: 0% (0/53) EG: 0% (0/27) CG: 0% (0/26)	None	Not reported

CG: control group; EG: experimental group; N: sample.

**Table 2:** Sensitivity analysis (leave-one-out) for the face-to-face intervention subgroup.

Study Omitted	OR (95% CI)	*p-*Value	I^2^
Dolbeault 2009 [24]	1.15 [0.45; 2.92]	0.7708	0%
Johns 2020 [30]	0.80 [0.44; 1.46]	0.4717	0%
Kimman 2011 [23]	0.71 [0.37; 1.34]	0.2909	0%
Omidi 2020 [29]	0.72 [0.39; 1.35]	0.3052	0%
Subgroup effect	0.79 [0.44; 1.41]	0.4196	0%

**Table 3:** Sensitivity analysis (leave-one-out) for the online intervention subgroup.

Study Omitted	OR (95% CI)	*p*-Value	I^2^
Admiraal 2017 [22]	1.10 [0.46; 2.63]	0.8314	11.2%
Çinar 2021 [27]	0.95 [0.45; 1.98]	0.8808	18.9%
Lee 2014 [28]	0.94 [0.45; 1.96]	0.8671	18.8%
Martínez-Miranda 2024 [26]	1.15 [0.57; 2.32]	0.7011	0%
Omidi 2020 [29]	0.79 [0.42; 1.51]	0.4814	0%
Smith 2019 [21]	0.74 [0.37; 1.48]	0.3997	0%
Subgroup effect	0.90 [0.49; 1.68]	0.7482	0%

**Table 4:** Quantiles of the posterior distribution for Odds Ratios of adherence versus usual care.

Modalities	2.5%	25%	50%	75%	97.5%
Face-to-face	0.34	0.57	0.74	0.96	1.71
Mixed	0.04	0.18	0.34	0.65	2.13
Online	0.48	0.77	0.98	1.27	2.19
Web-based	0.11	0.28	0.43	0.66	1.73

**Table 5:** League table of Odds Ratios between different intervention modalities.

	Face-to-face	Mixed	Online	Telephonic	Usual Care
Face-to-face	Face-to-face	0.46 (0.04, 3.37)	1.33 (0.46, 3.87)	0.58 (0.14, 2.23)	1.34 (0.58, 2.98)
Mixed	2.17 (0.3, 22.43)	Mixed	2.91 (0.41, 28.99)	1.28 (0.12, 16.48)	2.9 (0.47, 26.42)
Online	0.75 (0.26, 2.16)	0.34 (0.03, 2.46)	Online	0.43 (0.09, 2.04)	1.02 (0.46, 2.1)
Telephonic	1.74 (0.45, 7.02)	0.78 (0.06, 8.23)	2.31 (0.49, 11.22)	Telephonic	2.34 (0.58, 9.23)
Usual care	0.74 (0.34, 1.71)	0.34 (0.04, 2.13)	0.98 (0.48, 2.19)	0.43 (0.11, 1.73)	Usual care

**Table 6:** Ranking of interventions based on SUCRA values.

Position	Treatment	SUCRA Value
1	Usual care	0.767
2	Online	0.731
3	Face-to-face	0.519
4	Telephonic	0.254
5	Mixed	0.230

## Data Availability

No new data were created or analyzed in this study. Data sharing is not applicable to this article.

## Supplementary Materials

The following supporting information can be downloaded at: https://www.mdpi.com/article/10.3390/healthcare14091179/s1. File S1: Detailed search strategy in PubMed; File S2. Results excluded by full-text.
