# Supplementary material for: The Comparative Effectiveness of Education Modalities on Patient Adherence in Breast Cancer Survivors: A Systematic Review and Network Meta-Analysis
Source: Healthcare (Basel). 2026 Apr 28;14(9):1179. doi: 10.3390/healthcare14091179 (PMC13164239; doi:10.3390/healthcare14091179)
Supplement: Supplementary file 1 [file healthcare-14-01179-s001.zip › healthcare-4216247-supplementary.pdf]

Supplementary File S1. Detailed search strategy in PubMed.

("Breast Neoplasms"[MeSH] OR "breast cancer") AND ("Health Education"[MeSH]) AND ("Quality of Life"[MeSH]) Filters: Randomized Controlled Trial

(("Breast Neoplasms"[MeSH Terms] OR "breast cancer"[All Fields]) AND "Health Education"[MeSH Terms] AND "Quality of Life"[MeSH Terms]) AND (randomizedcontrolledtrial[Filter])

116 resultados

Supplementary File S2. Results excluded by full-text.

Reference		Exclusion Criteria	
1.	Kwiatkowski F, et al., 2013	Intervention	The intervention combines education with other therapy.
2.	Rashwan ZI et al., 2024	Intervention	The intervention is not aimed at improving quality of life.
3.	Kwiatkowski F, et al., 2017	Intervention	The intervention combines education with other therapy.
4.	Ridner SH et al., 2020	Intervention	The intervention is not aimed at improving quality of life.
5.	Ashing & Miller, A.M., 2016	Outcomes	Adherence rates and dropouts are not reported correctly.
6.	Ashing, & George, 2020	Outcomes	Adherence rates and dropouts are not reported correctly.
7.	Juarez G et al., 2013	Outcomes	Adherence rates and dropouts are not reported correctly.
